# *Prunella vulgaris* Suppresses HG-Induced Vascular Inflammation via Nrf2/HO-1/eNOS Activation

**DOI:** 10.3390/ijms13011258

**Published:** 2012-01-23

**Authors:** Sun Mi Hwang, Yun Jung Lee, Jung Joo Yoon, So Min Lee, Jin Sook Kim, Dae Gill Kang, Ho Sub Lee

**Affiliations:** 1 College of Oriental Medicine and Professional Graduate School of Oriental Medicine, Wonkwang University, Shinyong-dong, Iksan, Jeonbuk 570-749, Korea; E-Mails: madam@wku.ac.kr (S.M.H.); shrons@wku.ac.kr (Y.J.L.); 2 Center for Bioanalysis, Division of Metrology for Quality of Life, Korea Research Institute of Standards and Science, Doryong-dong, Yuseong-gu, Daejeon 305-340, Korea; 3 Hanbang Body-fluid Research Center, Wonkwang University, Shinyong-dong, Iksan, Jeonbuk, 570-749, Korea; 4 Korea Institute of Oriental Medicine, Jeonmin-dong, Yusung-gu, Daejeon 305-811, Korea; E-Mails: jskim@kiom.re.kr (J.S.K.)

**Keywords:** *Prunella vulgaris*, inflammation, NF-κB, eNOS, Nrf2, atherosclerosis

## Abstract

Vascular inflammation is an important factor which can promote diabetic complications. In this study, the inhibitory effects of aqueous extract from *Prunella vulgaris* (APV) on high glucose (HG)-induced expression of cell adhesion molecules in human umbilical vein endothelial cells (HUVEC) are reported. APV decreased HG-induced expression of intercellular adhesion molecule-1 (ICAM-1), vascular cell adhesion molecule-1 (VCAM-1), and E-selectin. APV also dose-dependently inhibited HG-induced adhesion of HL-60 monocytic cells. APV suppressed p65 NF-κB activation in HG-treated cells. APV significantly inhibited the formation of intracellular reactive oxygen species (ROS). HG-stimulated HUVEC secreted gelatinases, however, APV inhibited it. APV induced Akt phosphorylation as well as activation of heme oxygenase-1 (HO-1), eNOS, and nuclear factor E2-related factor 2 (Nrf2), which may protect vascular inflammation caused by HG. In conclusion, APV exerts anti-inflammatory effect via inhibition of ROS/NF-κB pathway by inducing HO-1 and eNOS expression mediated by Nrf2, thereby suggesting that *Prunella vulgaris* may be a possible therapeutic approach to the inhibition of diabetic vascular diseases.

## 1. Introduction

The fact that diabetic patients have an increased risk of atherosclerotic vascular disease has been well documented. Endothelial dysfunction, a recently proposed risk factor for atherosclerosis, plays a key role in the pathogenesis of diabetic atherosclerosis [1]. The adhesion of monocytes to the endothelium followed by transmigration into the subendothelial space is a key event in the pathogenesis of atherosclerosis. This can be mediated by the interaction of specific adhesion molecules on vascular endothelial cells with their integrin counter receptors on monocytes. In particular, endothelial cells in human atherosclerotic lesions have been shown to over express adhesion molecules such as intercellular adhesion molecule-1 (ICAM-1), vascular cell adhesion molecule-1 (VCAM-1), and E-selectin [2]. Studies based on cultured endothelial cells have clearly shown that incubation of endothelial cells with high concentration of glucose leads to an overexpression of adhesion molecules, suggesting a possible pathogenic link between hyperglycemia and atherosclerosis in diabetes [3]. Crucial enzymes involved in this process are matrix metalloproteinase (MMP)-2 and -9, which are called gelatinases, and whose transcription is regulated by nuclear factor-kappa B (NF-κB) [4]. A number of antioxidants and free radical quenchers have also been shown to block the NF-κB activation. Thus, the centrality of the ROS/NF-κB pathway is recognized as a key mediator involved in the regulation of inflammatory responses for vascular diseases.

Nuclear factor erythroid 2-related factor 2 (Nrf2) is a redox-sensitive transcription factor that normally resides in the cytoplasm bound to Kelch-like ECH-associated protein (Keap)-1 [5]. Upon activation by oxidative stress, binds to the antioxidant response element (ARE) and activates transcription of ARE-regulated genes. ARE-regulated genes may contribute to the maintenance of redox homeostasis by serving as endogenous antioxidant systems through the action of proteins such as heme oxygenase-1 (HO-1), ferritin, glutathione peroxidase (GPx), NAD(P)H: quinone oxidoreductase, *etc.* [6,7]. Nitric oxide (NO) produced by endothelial NO synthase (eNOS) plays a protective physiological role in the vasculature. Thus, Nrf2 and eNOS signaling could be an interesting target for the prevention or therapy of cardiovascular disease [8].

*Prunella vulgaris* is a perennial herb that is widely distributed in Far East Asian countries throughout Korea, China and Japan. *Prunella vulgaris* has been used as a traditional medicine to reduce sore throat, alleviate fever and accelerate wound healing. In addition, dried flowered fruit-spike of *prunella vulgaris* has been used in Oriental medicine to treat hypertension and tuberculosis [9]. A range of compounds (phenolic acids-rosmarinic acid, caffeic acid, campherol, rutin, triterpenoids, tannins) has been identified in prunella vulgaris [10]. Thus, we examined the anti-inflammatory effects of an aqueous extract of *prunella vulgaris* (APV) on high glucose (HG)-induced vascular inflammation in primary cultured human umbilical vein endothelial cells (HUVEC).

## 2. Results and Discussion

**Figure 1:**
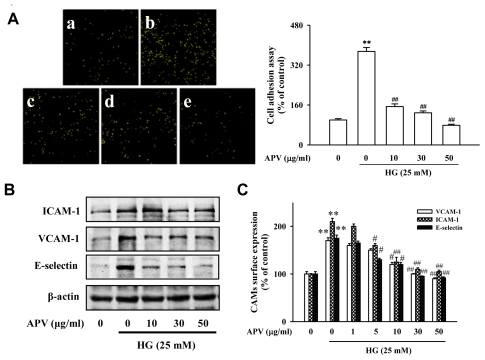
Aqueous extract from *Prunella vulgaris* (APV) inhibits high glucose (HG)-induced vascular inflammation. (**A**) Adhesion of fluorescence-labeled monocytic HL-60 cells were added to the monolayer human umbilical vein endothelial cells (HUVEC) and allowed to adhere for 1 h. a, Control; b, HG (25 mM); c, HG + APV (10 μg/mL); d, HG + APV (30 μg/mL); e, HG + APV (50 μg/mL), respectively. The amounts of adherent HL-60 cells were monitored by fluorescence microscopy; (**B**) Western blots of ICAM-1, VCAM-1, and E-selectin were detected as described in the Experimental Section. Each electrophoretogram is representative of the result from five individual experiments; (**C**) HUVEC surface expressions of intercellular adhesion molecule-1 (ICAM-1), vascular cell adhesion molecule-1 (VCAM-1), and E-selectin were analyzed by cell-based ELISA. Values are means ± S.E. of 6 independent experiments with triplicate dishes. ** *P* < 0.01, *vs.* control; # *P* < 0.05; ## *P* < 0.01, *vs.* HG alone.

One of the earliest events in atherogenesis is the adhesion of monocytes to the endothelium, followed by their infiltration and differentiation into macrophages [2]. These cell adhesion molecules primarily mediate the adhesion of monocytes specifically found in atherosclerosis lesions to the vascular endothelium. To explore the effect of APV on endothelial cell-leukocyte interaction, we examined the adhesion of HL-60 cells to HG-activated HUVEC under static conditions. Figure 1A showed that control HUVEC showed minimal binding to HL-60 cells, but adhesion was markedly increased when they were treated with HG. Pretreatment with 10–50 μg/mL APV significantly decreased the number of HL-60 cells adhering to HG-induced HUVEC (*P* < 0.01). In the experiment to determine whether APV inhibits HG-induced increase of ICAM-1, VCAM-1, and E-selectin expression, various APV concentrations ranging from 10 to 50 μg/mL were added to HUVEC. As shown in Figure 1B, pretreatment with APV decreased HG-induced ICAM-1, VCAM-1, and E-selectin expression with the inhibitory effect being observed over 5 μg/mL. The results using cell based-ELISA demonstrated that HG also increased ICAM-1, VCAM-1, and E-selectin levels. However, pretreatment with APV significantly decreased HG-induced ICAM-1, VCAM-1, and E-selectin levels (Figure 1C). In this study, APV (1–50 μg/mL) did not alter any cytotoxicity (data not shown).

ROS has been implicated as a common second messenger in various pathways leading to NF-κB activation [11]. To confirm that inhibitory effect of APV on HG-induced oxidative stress, HUVEC were labeled with a cell-permeable fluorescent dye, CM-H_2_DCFDA and analyzed by flow cytometry. DCF fluorescence level showed a significant increase after incubation with 25 mM glucose. However, pretreatment with APV (10–50 μg/mL) significantly inhibited HG-induced DCF-sensitive ROS levels (Suppl. 1A). Hydrogen peroxide assay showed APV statistically inhibited HG-induced hydrogen peroxide level (*P* < 0.01, Supplementary. 1B). Curcumin or NAC, well known anti-oxidants, blocked HG-induced hydrogen peroxide level, respectively (*P* < 0.01, *P* < 0.05). Thus, this result suggested that the anti-oxidant property of APV leads to inhibition of vascular inflammation in high glucose condition. In Western blotting analysis, NF-κB p65 nuclear protein level was significantly increased by HG. However, pretreatment of APV inhibited NF-κB expression into the nucleus in a dose-dependent manner. In addition, HG-activated HUVEC exhibited marked decrease in IκB-α level and increase in phospho-IκB-α level. Furthermore, APV blocked HG-induced IκB-α phosphorylation to activate NF-κB (Supplementary 2A). To confirm consistency with the Western blotting results, immunocytochemistry was performed using a p65 NF-κB and FITC-conjugated antibody. HG increased green signal of p65 NF-κB expression in nucleus, APV decreased the HG-induced p65 NF-κB expression in nucleus (Supplementary 2B).

**Figure 3:**
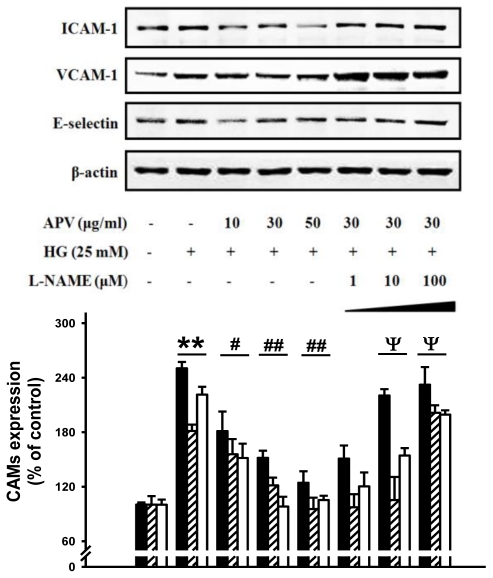
Effect of an APV on HG-induced cell adhesion molecules expression in the presence or absence of L-NAME. HUVECs were preincubated with L-NAME (1~100 μM) for 30 min prior to the treatment of APV, followed by HG for 24 h. The lower panel depicts quantitative data, expressed as ICAM-1 (■), VCAM-1 (▨), and E-selectin (□) normalized to β-actin, and the results are expressed as the % of the control. Each electrophoretogram is representative of the results from five individual experiments. ** *P* < 0.01 *vs.* control, # *P* < 0.05, ## *P* < 0.01 *vs.* HG alone, ψ *P* < 0.05 *vs.* APV + HG.

**Figure 4:**
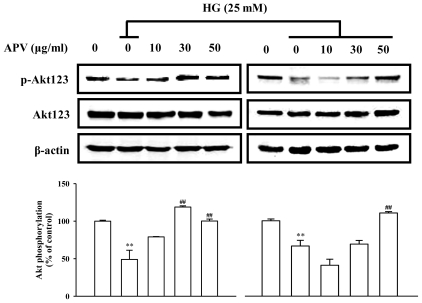
Effect of APV on Akt phosphorylation. HUVEC were treated with APV or HG alone for 1 h (left), and pretreated with APV for 30 min and then stimulated with HG for 1 h (right). Each electrophoretogram is representative of the results from five individual experiments. ** *P* < 0.01, *vs.* control; ## *P* < 0.01, *vs.* HG alone.

**Figure 5:**
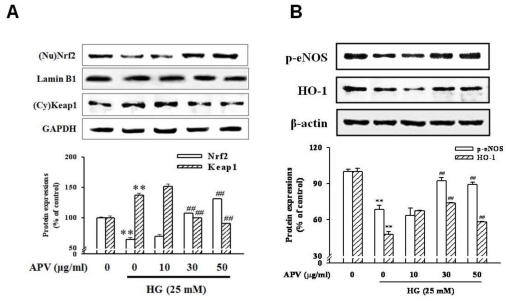
Effect of APV on Nrf2, eNOS, and HO-1 activation. (**A**) HUVEC were pretreated with APV for 30 min and then stimulated with HG for 1 h. The nuclear and total cellular protein extracts were blotted with Nrf2, lamin B1, Keap1, GAPDH, respectively. Nuclear Nrf2 levels were normalized to lamin B1 levels; (**B**) The total protein (40 μg) extracts were blotted with the antibodies specific for eNOS Ser-1177, HO-1, and β-actin. Each electrophoretogram is representative of the results from five individual experiments. ** *P* < 0.01, *vs.* control; ## *P* < 0.01, *vs.* HG alone.

We hypothesized that APV activate endogenous protective pathways, if a defense system against HG-induced vascular inflammation exists. Since reduced NO-cGMP signaling contributes to vascular inflammation, firstly, endothelial-derived NO production has attracted increasing attention in this study [12]. In experiments to determine whether APV mediated NO signaling involved in vascular protection against the HG-induced vascular inflammation, HUVECs were treated with L-NAME, N(G)-nitro-L-arginine methyl ester, prior to the APV treatment. After exposure to HG, cell adhesion molecules expression was measured by Western blotting. Figure 3 showed that L-NAME attenuated the protective effect of APV in HG-induced ICAM-1, VCAM-1, and E-selectin expressions, suggesting a vascular protective role of eNOS-NO signaling in HG-induced vascular inflammation. Previously, it was also reported that *Buddleja officinalis* exerts anti-vascular inflammatory property via NO signaling activation [13]. Secondly, the Nrf2/anti-oxidant response element (ARE) pathway plays an important role in regulating cellular anti-oxidants, including HO-1, cytoprotective enzyme. Essential Role of Nrf2-mediated HO-1 upregulation was reported in adaptive survival response to oxidative stress [14]. To determine that APV activates Akt/Nrf2-mediating NO signaling to contribute to the anti-inflammatory process, we investigated the effects of APV on Akt phosphorylation. Confluent HUVEC were treated with APV in absence or presence of HG. APV significantly induced Akt phosphorylation over 30 μg/mL concentration without HG stimulation. HG significantly decreased Akt phosphorylation, however, pretreatment with APV recovered Akt phosphorylation (Figure 4). In addition, HG decreased Nrf2 expression in nuclear fraction, pretreatment with APV significantly attenuated in a dose-dependent manner (*P* < 0.01). The concentration of keap1 in the cytosol was decreased concomitantly by APV concentration (*P* < 0.01, Figure 5A). Figure 5B showed that HG decreased both Ser-1177 eNOS phosphorylation and HO-1 induction. Pretreatment with APV significantly attenuated HG-induced decrease of eNOS phosphorylation and HO-1 induction in a dose-dependent manner.

Under HG condition, APV increased Akt phosphorylation and HO-1 expression in a dose-dependent manner. Activation of Nrf2 leads to a reduction of ROS, to elevated levels of NO, and to a transient reduction of eNOS protein levels in primary human endothelial cells [15]. In the present study, APV showed anti-oxidant property and Nrf2 activation, whereas, HG-induced decrease of eNOS phosphorylation was recovered by pretreatment with APV. It suspected that this discrepancy may be due to the distinction between time and concentration, even though eNOS plays a protective physiological role in the vasculature. Recently, nitrosative stress caused by reactive nitrogen species such as nitric oxide and peroxynitrite overproduced during inflammation leads to cell death and has been implicated in the pathogenesis of many human aliments. However, relatively mild nitrosative stress may improve cellular defense capacities, rendering cells tolerant or adaptive to ongoing and subsequent cytotoxic challenges [16]. The regulating mechanism of NO in vascular inflammation remains unclear. *In vivo* upregulation of eNOS gene expression while maintaining eNOS activity seems to be a reasonable and realistic strategy for preventing cardiovascular disease [17]. In addition, the fact that activated PI-3-kinase/Akt-Nrf2 signaling also plays a critical role in the regulation of the vasoprotective eNOS supported our results. It is clear that HO-1 has been shown to be an important biological target of NO. In addition, NO can induce HO-1 expression and interleukin-8 production, particularly, in vascular endothelial cells [18]. Thus, though we cannot rule out biphasic effect of bioactive NO, APV activated Nrf2-mediated eNOS/HO-1 pathways contribute to defense against HG-induced vascular inflammation. Further study is needed to investigate a possible mechanism of eNOS/HO-1-mediated cell adhesion molecules deactivation by APV in HUVEC.

## 3. Experimental Section

### 3.1. Preparation of an Aqueous Extract of *Prunella vulgaris var. Lilacina*

The *Prunella vulgaris var. lilacina* were purchased from an Herbal Medicine Co-operative association in Jeonbuk Province, South Korea in January, 2010. A voucher specimen (No. HBN161) has been deposited in the Herbarium of the Professional Graduate School of Oriental Medicine, Wonkang University (Korea). The dried *Prunella vulgaris var. lilacina* (100 g) was soaked for 2 h in water (1 L) and then boiled in distilled water at 100 °C for 2 h. The yield of aqueous extract of *Prunella vulgaris var. lilacina* (APV) was approximately 16.27% of the plant powder. The extract was subsequently concentrated using rotary evaporator and then used in the present study.

### 3.2. Cell Culture

Primary cultured HUVEC and endothelial cell growth medium (EGM-2) containing 2.5% fetal bovine serum (FBS) and growth supplements were purchased from Cambrex (East Rutherford, NJ, USA). HUVEC which were used between passages 3 and 8 were maintained in EGM-2 in a humidified chamber containing 5% CO_2_ at 37 °C.

### 3.3. Cell Enzyme Linked Immunosorbent Assay (ELISA)

ELISA was used to determine the level of ICAM-1, VCAM-1, and E-selectin expression on the cell surface, as previously described with minor modifications. Briefly, HUVEC were fixed by 1% paraformaldehyde and exposed to mouse anti-human ICAM-1, VCAM-1, or E-selectin antibodies at 1:1000 dilution in the phosphate-buffered saline (PBS) containing 1% bovine serum albumin (BSA) for 2 h at room temperature. The cells were washed and incubated with a horseradish peroxidase (HRP)-conjugated secondary antibody. The expression of VCAM-1, ICAM-1, or E-selectin was quantified by adding a peroxidase substrate solution (40 mg *o*-phenylenediamine and 10 μL 30% H_2_O_2_ in 100 mL 0.05 M citrate-phosphate buffer). After incubation for 30 min at 37 °C, the reaction was stopped by addition of 5 N H_2_SO_4_, and the absorbance of each well was measured at 490 nm by a Multiskan microplate reader (Thermo LabSystems Inc., Franklin, MA, USA).

### 3.4. Monocyte-Endothelial Cell Adhesion Assay

The cell adhesion assay was modified as described. Briefly, regularly passaged HL-60 cells were labeled with 10 μg/mL 2′,7′-bis-(carboxyethyl)-5,6-carboxyfluorescein acetoxymethyl ester (BCECF/AM, Sigma Chemical Co., St. Louis, MO, USA) at 10 μM final concentration in RPMI-1640 medium containing 10% FBS at 37 °C for 30 min. The labeled cells were harvested by centrifugation and washed three times with PBS before suspension in the medium, and added to HUVEC in six-well culture plates at 4 × 10^5^ cells/mL. The co-incubation was done at 37 °C for 1 h and nonadhering HL-60 cells (American Type Culture Collection, Manassas, VA, USA) were removed by stringent washing two times with PBS. HL-60 cells bound to HUVEC were measured by fluorescence microscopy (Leica DMIRB, Leica, Germany) and were lysed with 50 mM Tris-HCl, pH 8.0, containing 0.1% sodium dodecyl sulfate (SDS). The fluorescent intensity was measured using a spectrofluorometer (F-2500, Hitachi, Tokyo, Japan) at an excitation and emission wavelength of 485 nm and 535 nm, respectively. The adhesion data are represented in terms of the percentage change compared with the control values.

### 3.5. Preparation of Cytoplasmic and Nucleus Extracts

The cells were rapidly harvested by sedimentation and nuclear and cytoplasmic extracts were prepared on ice as previously described by the method of Mackman *et al.* [19]. Cells were harvested and washed with 1 mL buffer A (10 mM HEPES, pH 7.9, 1.5 mM MgCl_2_, 19 mM KCl) for 5 min at 600 g. The cells were then resuspended in buffer A and 0.1% NP 40, left for 10 min on ice to lyse the cells and then centrifuged at 600 g for 3 min. The supernatant was saved as cytosolic extract. The nuclear pellet was then washed in 1 mL buffer A at 4200 g for 3 min, resuspended in 30 μL buffer C (20 mM HEPES, pH 7.9, 25% glycerol, 0.42 M NaCl, 1.5 mM MgCl_2_, 0.2 mM EDTA), rotated for 30 min at 4 °C, then centrifuged at 14,300 g for 20 min. The supernatant was used as nucleus extract. The nucleus and cytosolic extracts were then analyzed for protein content using Bradford assay. There were no contamination between nuclear and cytoplasmic fractions by Glyceraldehyde-3-phosphate dehydrogenase (GAPDH) in cytoplasmic fraction, and lamin B1 for nuclear fraction loading control.

### 3.6. Western Blot Analysis

Cell homogenates (40 μg of protein) were separated on 10% SDS-polyacrylamide gel electrophoresis and transferred to nitrocellulose paper. Blots were then washed with H_2_O, blocked with 5% skimmed milk powder in Tris-Buffered Saline Tween-20 (TBST) (10 mM Tris-HCl, pH 7.6, 150 mM NaCl, 0.05% Tween-20) for 1 h, and incubated with the appropriate primary antibody at dilutions recommended by the supplier. Then the membrane was washed and primary antibodies were detected with goat anti-rabbit-IgG or rabbit anti-mouse-IgG conjugated to horseradish peroxidase, and the bands were visualized with enhanced chemiluminescence (Amersham Bioscience, Buckinghamshire, UK). Protein expression levels were determined by analyzing the signals captured on the nitrocellulose membranes using the ChemiDoc image analyzer (Bio-Rad Laboratories, Hercules, CA, USA).

### 3.7. Gelatin Zymography

MMP-2 and MMP-9 enzymatic activities were assayed by gelatin zymography. Samples were electrophoresed on a 1 mg/mL gelatin containing 10% SDS-polyacrylamide gel. After electrophoresis, the gel was washed twice with washing buffer (50 mM Tris–HCl, pH 7.5, 100 mM NaCl, 2.5% Triton X-100), followed by a brief rinsing in washing buffer without Triton X-100. The gel was incubated with incubation buffer (50 mM Tris–HCl, pH 7.5, 150 mM NaCl, 10 mM CaCl_2_, 0.02% NaN_3_, 1 μM ZnCl_2_) at 37 °C. After incubation, the gel was stained with Commassie brilliant blue R-250 and destained. A clear zone of gelatin digestion was represented with the MMP activity.

### 3.8. Statistical Analysis

Data are expressed as a mean ± S.E., and the data were analyzed using one-way ANOVA followed by a Dunnett’s test or Student’s *t* test to determine any significant differences. A *P* value < 0.05 was considered significant.

## 4. Conclusion

**Figure 6:**
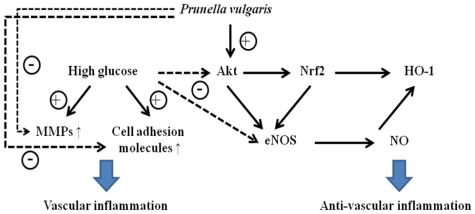
A scheme for the inhibitory effect of APV on HG-induced vascular inflammation. This simplified scheme depicts HG induced MMPs and cellular adhesion molecules. ROS/NF-κB signaling is a cellular event involved in development of vascular inflammation. On the other hand, APV induces Akt phosphorylation, it activates Nrf2 or eNOS/NO, and finally activates HO-1. Nrf2 also activates eNOS/NO signaling pathway.

Vascular inflammation induced by HG occurs early in the development of atherosclerosis, and determines future diabetic complications. The present study suggested that APV significantly suppressed the following events in cultured vascular endothelial cells: HG-induced intracellular ROS formation and redox-sensitive NF-κB activation via suppression of IκB degradation and phosphorylation; cell adhesion molecules expression; adhesion to monocytes; and MMP-2/-9 proteolytic activities. Simultaneously, APV activates vascular protective signal pathways against HG-induced vascular inflammation in the following manner: Firstly, APV-induced PI3K/Akt-mediated Nrf2 activation, via the suppression Keap1 degradation, could induce activation of HO-1 and eNOS (Figure 6). Thus, APV inhibits *in vitro* vascular inflammation and may prevent diabetic atherosclerosis via inhibition of ROS/NF-κB pathway by inducing Nrf2-mediated HO-1 and eNOS activation. This data casts a new light on the actions of *Prunella vulgaris* and its potential benefits to diabetic patients for preventing diabetic vascular complications.

## Supplementary Material



## Figures and Tables

**Figure 2:**
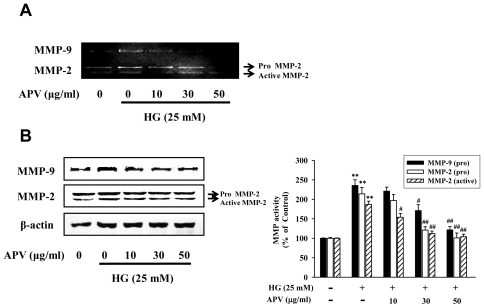
Effect of APV on HG-induced gelatinase activity. (**A**) The level of matrix metalloproteinases (MMPs) in conditioned supernatant was prepared, and gelatin zymography was performed; (**B**) The cell lysates were examined for the expression of MMPs by Western blotting. Each electrophoretogram is representative of the results from five individual experiments.
