# Supplementary material for: Prunella vulgaris Suppresses HG-Induced Vascular Inflammation via Nrf2/HO-1/eNOS Activation
Source: Int J Mol Sci. 2012 Jan 23;13(1):1258–68. doi: 10.3390/ijms13011258 (PMC3269750; doi:10.3390/ijms13011258)
Supplement: Supplementary file 1 [file ijms-13-01258-s001.pdf]

Supplementary Material

1. Methods

1.1. Intracellular ROS Production Assay

The fluorescent probe 5-(and-6)-chloromethyl-2',7'-dichlorodihydro-fluorescein diacetate, acetyl ester (CM-H_2DCFDA) was used to determine the intracellular generation of ROS by HG described elsewhere. Briefly, the confluent HUVEC in the 24-well plates were pretreated with APV for 1 h. After removing the APV from the wells, the cells were incubated with 5 μM CM-H_2DCFDA for 30 min. The cells were then stimulated with 25 mM glucose for 1 h, and the fluorescence intensity was measured by flow cytometry on FACScalibur (BD, San Diego, CA) and spectrofluorometer (F-2500, Hitachi, Tokyo, Japan).

1.2. H_2O_2 Release

The levels of H_2O_2 were determined by a modification of the method of Zhou *et al* [1]. The cells washed twice with ice-cold PBS and harvested cells by microcentrifugation were resuspended in a Krebs-Ringer phosphate solution. One hundred microliter of the reaction mixture (50 μM Amplex Red reagent containing 0.1 U/mL HRP in KRPG) was added into each microplate well and then prewarm at 37 °C for 10 min. After then, reaction was started as adding resuspended cells in 20 μL of KRPG. Fluorescence readings became stable within 30 min of starting of reaction. The fluorescence intensities of reaction mixtures were measured at 30 min using fluorescence microplate reader (Multiskan, Thermo labsystems Inc, Franklin, MA) equipped for absorbance at ~560 nm.

1.3. Immunofluorescence Microscopy

HUVEC were seeded on coverslip and incubated for 24 h. HUVEC were treated with APV for 1 h, then 25 mM glucose was added, and the incubation continued for an additional 1 h. After several washes with PBS, cells were fixed with 4% paraformaldehyde for 30 min at room temperature. It was permeabilized by treatment with 0.1% Triton X-100 for 30 min at room temperature. The cells were overlaid with protease-free BSA for 30 min, rinsed with PBS and incubated with primary antibodies (p65 or Nrf2, 1:100 in PBS) in a humid chamber overnight at 4 °C. They were gently washed several times with PBS before incubation with secondary antibody (goat anti-rabbit IgG conjugated with FITC, 1:128 in PBS) for 2 h. Cells were finally washed three times with PBS, coverslips were mounted with Dako Fluorescent mounting medium onto glass slides, and examined under a fluorescence microscope (Axiovision 4, Zeiss, Germany).

Supplement 1. APV inhibits HG-induced ROS formation. (A) Cells were pretreated with APV (10–50 µg/mL) for 30 min and then stimulated with HG for 1 h. CM-H_2DCFDA (20 µM) was added for 30 min and ROS formation was measured by FACS-analysis. (B) H_2O_2 assay was measured in a 96-well plate reader and % induction was calculated in relation to unstimulated HUVEC. Cur, curcumin (10 µM); NAC, *N*-acetylcysteine (10 mM). Values are means ± S.E. of 6 independent experiments with triplicate dishes. $*p < 0.01$ vs. control; # $p < 0.01$ vs. TNF-α alone.

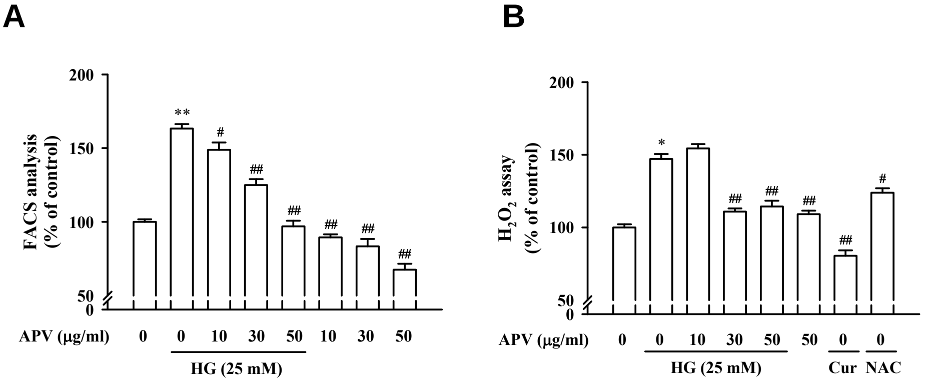

Supplement 2. APV inhifbits HG-induced NF-κB activation. (A) HUVEC were pretreated with APV (10–50 µg/mL) for 30 min and then stimulated with HG for 1 h. Nuclear (Nu) and cytoplasmic (Cy) fractions were extracted and protein levels determined by western blot analysis. (B) NF-κB p65 translocation into nucleus (green) was visualized by indirect fluorescence staining. Each electrophoretogram is representative of the results from five individual experiments.

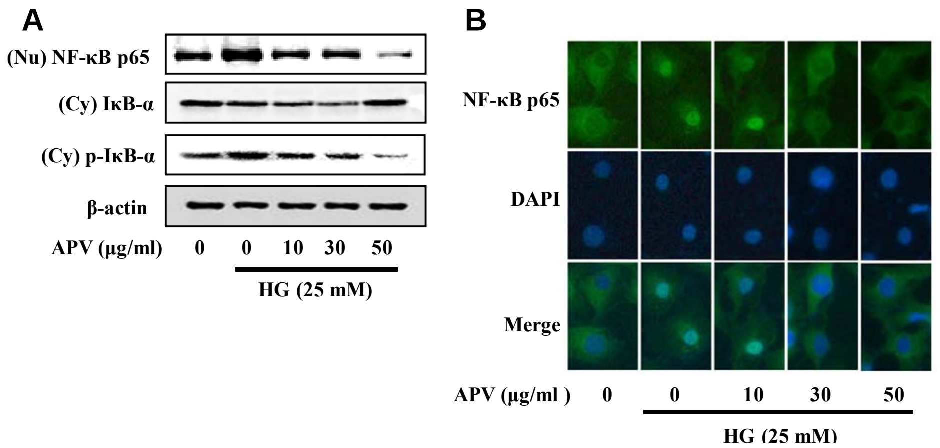

1. Zhou, M.; Diwu, Z.; Panchuk-Voloshina, N.; Haugland, R.P. A stable nonfluorescent derivative of resorufin for the fluorometric determination of trace hydrogen peroxide: applications in detecting the activity of phagocyte NADPH oxidase and other oxidases. *Anal. Biochem.* **1997**, *253*, 162–168.

© 2012 by the authors; licensee MDPI, Basel, Switzerland. This article is an open access article distributed under the terms and conditions of the Creative Commons Attribution license (http://creativecommons.org/licenses/by/3.0/).
